# Effect of incorporating wing veins on soft wings for flapping micro air vehicles

**DOI:** 10.3389/frobt.2023.1243238

**Published:** 2023-08-07

**Authors:** Risa Ishiguro, Takumi Kawasetsu, Koh Hosoda

**Affiliations:** Adaptive Robotics Laboratory, Graduate School of Engineering Science, Osaka University, Toyonaka, Japan

**Keywords:** bio-inspired flying vehicle, soft wing, micro air vehicle, wing vein, FWMAV

## Abstract

Small insects with flapping wings, such as bees and flies, have flexible wings with veins, and their compliant motion enhances flight efficiency and robustness. This study investigated the effects of integrating wing veins into soft wings for micro-flapping aerial vehicles. Prototypes of soft wings, featuring various wing areas and vein patterns in both the wing-chord and wing-span directions, were fabricated and evaluated to determine the force generated through flapping. The results indicated that the force is not solely dependent upon the wing area and is influenced by the wing vein pattern. Wings incorporating wing-chord veins produced more force compared to those with wing-span veins. In contrast, when the wing area was the specific wing area, wings with crossed wing veins, comprising both wing-span veins and wing-chord veins, produced more force. Although wing-chord veins tended to exert more influence on the force generated than the wing-span veins, the findings suggested that a combination of wing-span and wing-chord veins may be requisite, depending upon the wing area.

## 1 Introduction

The field of micro air vehicles (MAVs) that emulate the flapping wing motion of insects and birds has been rapidly advancing. In particular, flapping MAVs have been extensively studied (Dileo and Deng, 2009; Ma et al., 2013; Liu et al., 2016; Zou et al., 2016; Chen et al., 2019) with the aim of designing air vehicles that are smaller than their fixed-wing and rotary-wing counterparts (Ma et al., 2013). Flapping MAVs are considered to be safer than rotary-wing MAVs because of the probability of causing damage to themselves upon contact with other objects or humans is significantly less. MAVs can be employed to measure terrain and search for victims. Smaller MAVs have the potential to expand applications in environments characterized by various obstacles, such as forests and buildings, where the available flight space is limited.

Wings are the largest component of these vehicles. Therefore, to miniaturize the flapping wings, it is crucial to reduce the wing size while maintaining flight performance using the same actuators. For a simple rigid plate wing, reducing the wing size results in decreased output power, necessitating an increase in the flapping frequency and thus requiring increased actuator power. However, for motors, the most mainstream actuators employed in flapping MAVs (Dileo and Deng, 2009), increasing the output power leads to an increase in the size and mass, demanding more power for flight. More compact, high-power actuators, such as piezoelectric bimorphs and dielectric elastomers, have been developed for flapping robots (Ma et al., 2013; Chen et al., 2019). Nevertheless, these alternatives fall short of motors in terms of availability and ease of operation. Consequently, there is a need for a method that enables wing size reduction without compromising power output from flapping motion while using the same actuator.

Wing veins could potentially hold the key to reducing wing size while maintaining the power output of flapping wings. The small, high-performance insect wings possess wing veins, which are reinforcing structures that vary in design across species. These veins enhance wing stiffness (Wootton, 1992; Combes and Daniel, 2003), inhibit crack propagation (Dirks and Taylor, 2012; Rajabi et al., 2015; Zhang et al., 2018), and may be associated with the forces generated by wing flapping (Ennos, 1988; Mountcastle and Combes, 2013). Experiments conducted using wings with only wing veins and membranes on the leading edge have demonstrated that if the membrane stiffness is too low, the force generated by wing flapping will be small (Ryu et al., 2019). Consequently, wing veins that increase wing stiffness are considered an effective means of maintaining the force produced by wing flapping. Understanding the relationship between wing veins and flight performance makes it possible to optimize wing vein design to minimize wing size with reduced power loss, even when using simple wing flapping.

Instead of replicating the intricate wing veins of insects, a simplified wing vein design could be employed to approach an ideal configuration for flapping MAVs. Evidently, empirical evidence suggest that simplified wing veins can be more effective than complex insect-like wing veins. Majority of wings developed for flapping MAVs so far have featured simplified wing veins, typically consisting of two oblique straight lines (Dileo and Deng, 2009; Ma et al., 2013; Liu et al., 2016; Zou et al., 2016; Chen et al., 2019). However, there is a lack of published data comparing the relationship between wing veins and the force produced by wing flapping, thus resulting in limited understanding of this relationship. Moreover, investigating the forces generated by the flapping of flexible wings through numerical simulations that combine actively moving elastic bodies and fluids is not easy. For an improved understanding, it is necessary to conduct flapping experiments in real-world environments.

In this study, we focused on wing veins oriented in the wing-chord and wing-span directions and investigated the relationship between these veins, variations in the wing area, and the force generated by wing flapping. Artificial wings with different veins configurations and wing areas were fabricated, and the force produced by a simple flap of the wings was measured. To focus on the performance of the simplest flapping robot resulting from wing flexibility, we conducted experiments while fixing the wing roots to prevent pitch rotation, same as some previous flapping robot studies (De Croon et al., 2009; Gerdes et al., 2014; Sukvichai and Yajai, 2020). The results showed that the force generated by wing flapping was not proportional to wing area, and wings with veins only in the wing-chord direction produced more force than those with veins solely in the wing-span direction, except when the wing area was a specific wing area. In that case, the crossed wing veins incorporating both wing-span and wing-chord directions, generated greater force.

## 2 Materials and methods

### 2.1 Artificial wings with wing veins

**FIGURE 1:**
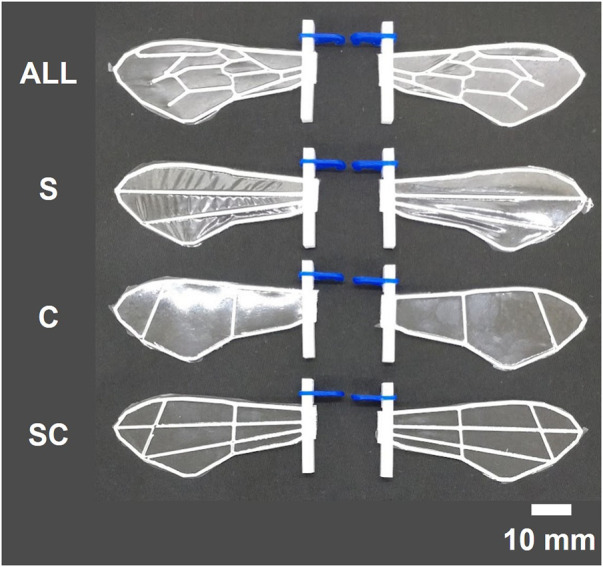
Photographs of the wings with various wing veins produced in this study. From top to bottom: wings with all veins types (ALL), simplified wing-chord directional veins(S), simplified wing-span directional veins (C), and crossed wing veins (SC). All wings are of the standard size.

**FIGURE 2:**
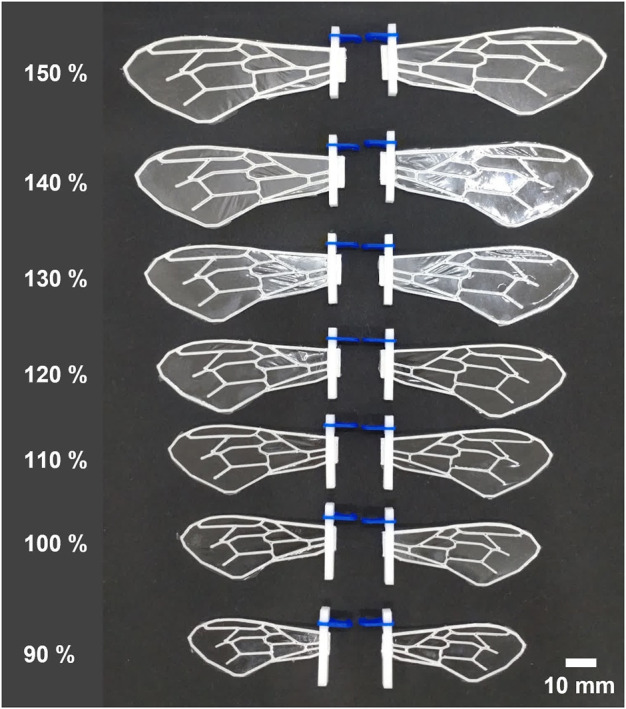
Photographs of wings with ALL types of wing veins produced in this study with various sizes. The percentages values on the left of each wing indicate the percentage of length relative to the standard wing size.

To investigate the variations in force produced by the same wing flap when wing veins and wing area are altered, we fabricated a total of 28 different artificial wings, encompassing four different vein designs and seven unique wing areas (Figures 1, 2). We created four types of wing veins: one that mimics the wings of the honeybee (*Apis mellifera*) as referenced in Angel-Beamonte et al. (2018) represented as ALL; one with only two veins in the wing-chord direction (S); one with only two veins in the wing-span direction (C); and one with a crossed type (SC) that combines the S- and C-types (Figure 1). The outer frames of all the wings are consistent with the outer frames of the ALL wing type. We fabricated wings of different areas by varying the wing-span and wing-chord in seven different sizes (90%, 100%, 110%, 120%, 130%, 140%, 150%), based on a reference wing with a wing-span of 57.254 mm and a wing-chord of 24.381 mm used in our preliminary experiment (Ishiguro et al., 2021), as represented in Figure 2. A list of the produced wing areas and their length ratio values relative to the reference wings are shown in Table 1. All wing veins, including the leading edge, had rectangular cross-sections. The wing vein widths of the reference wings were all set at 0.8 mm. The leading edge of the wing was thicker than the rest of the wing, with the remaining wing thickness being half of that of the leading edge. The thickness of the wing’s leading edge was determined for each size using the method described in the following sections. The minimum wing area was determined to be approximately 773 mm^2^ owing to the lower limit of the number of layers that could be fabricated. The tip-to-tip length of the largest wing used in the experiment was approximately 190 mm when attached to the flapping device. This is similar to that of *Megaloprepus caerulatus*, the largest species of dragonfly with horizontally elongated, non-folded wings, which is a larger insect than the bee used as a reference for wing veins in this study.

**TABLE 1:** Wing size and layer number.

Percentage for standard wings [%]	Wing-span length [mm]	Wing-chord length [mm]	Leading edge thickness [mm]	Leading edge layer number	Wing area [mm^2^]	Wing-span inertia *I*_*xx*_ [mm^4^]	Wing-span bending stiffness *EI*_*xx*_ [ N ⋅ mm^2^]
90	51.088	21.943	0.356	3	773.058	0.003	5.956
100	57.254	24.381	0.534	4	954.392	0.010	22.333
110	63.419	26.820	0.712	4	1154.814	0.026	58.232
120	69.585	29.258	0.890	5	1374.324	0.056	124.075
130	75.750	31.696	1.068	6	1612.922	0.106	232.268
140	81.915	34.134	1.245	7	1870.608	0.180	396.248
150	88.081	36.572	1.424	8	2147.382	0.289	635.262

**FIGURE 3:**
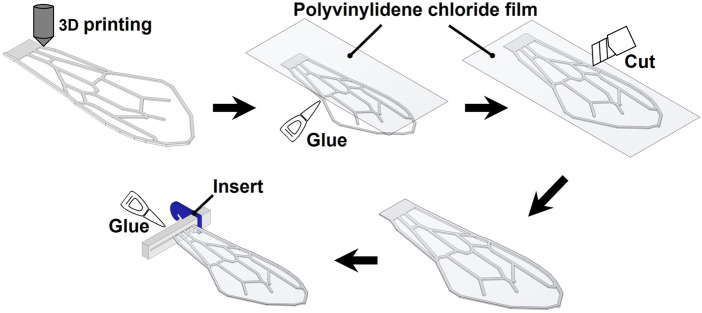
Wing manufacturing methods.

Figure 3 illustrates the wing fabrication procedure. An FDM 3D printer (Stratasys F120; Stratasys Ltd.) was used to create the artificial wing veins. Because the minimum build thickness of the 3D printer was 0.1778 mm (0.007 inches), the thickness of the wing veins for the artificial wings fabricated in this study was an integral multiple of this thickness. We used acrylonitrile styrene acrylate (ASA) as the 3D printing material. Young’s modulus (*E*) and density (*ρ*) of this material were 2.2 × 10^3^ MPa and 1.047 × 10^3^ kg/m^3^, respectively. After printing the wing veins, a thin film of polyvinylidene chloride was applied to the flat leading edge using adhesive, and the excess film extending beyond the wing outline was removed using a cutter knife. Parts for attaching the flapping device, were attached to the base of the wing using glue, as described below.

The thickness of the leading edge of the wings was adjusted so that when the wings were attached to the flapping device, the wing exhibited the primary mode of flapping in wing-span direction, akin to that observed in real insects. The mode of flapping was determined by evaluating the relationship between the frequency of the flapping wings and the device. When the natural frequency of the wings is greater than the flapping frequency of the device, the flapping became the primary mode. In contrast, when the natural frequency is lower than the flapping frequency of the device, the flapping became a secondary or higher mode. When wing flapping occurred in a secondary mode, the volume of air displaced by the wings was smaller than that in the primary mode; thereby generating a smaller force. The wing-span bending stiffness in insects is known to be highly dependent on the thickness of the leading edge of the wing (Combes and Daniel, 2003). Further, the wing bending stiffness can be modeled as that of a cantilever beam (Chen et al., 2016). The influence of the mode in the chord direction on the mode in the span direction is minimal. We did not consider the wing-chord natural frequencies here because our goal was to make the deformation mode in the wing-span direction the primary mode resemble that of insects. Therefore, we modeled the wing-span bending stiffness and the natural frequency of the entire wing by considering them as equivalent to those exhibited by the leading edge of the wing. In this case, where the wing-span length is *l*, the thickness of the leading edge of the wing is *h*, and the width is *b*, the wing-span bending stiffness (*EI*_*xx*_) and the primary natural frequency (*f*) are

$$EI_{xx}=E\times \frac{bh^{3}}{12},$$ (1)

$$f=\frac{1}{2\pi }\frac{1.875^{2}}{l^{2}}\sqrt{\frac{E}{\rho hb}\frac{bh^{3}}{12}},$$ (2)

Here, *I*_*xx*_ denotes wing-span inertia. The Young’s modulus *E* and density *ρ* of the material used here have been described earlier in this section.

In this study, to eliminate the effects of mode variation, the thickness of the wings for each size was determined to ensure that the flapping of all the wings of all sizes occur in primary mode and nearly identical primary natural frequencies. This operation results in nearly identical values of bending stiffness in the wing-span direction, and thus wing-span inertia *I*_*xx*_, for all wings. The flapping device flapped the wings at approximately 32 Hz, as described in subsequent section. Based on Eq. 2, we specified the wing thickness so that the primary natural frequency (*f*) was 1.25 times higher than the frequency of the flapping device. Furthermore, we calculated the thickness (*h*) at which the primary natural frequency was 40 Hz and selected the closest thickness for the leading edge out of the multiple variations of one layer of 0.1744 mm obtained from the 3D printer. Because inertia affects wing bendability, this manipulation implies that, inertia was manipulated to align wing bendability for all wing sizes. The values of wing-span inertia and bending stiffness calculated by the above method are shown in Table 1. Because of the root constraint of the wing, the wing-chord primary natural frequency cannot be calculated as simply as the wing-span resonant frequency. Therefore, it is difficult to predict the deformation in the wing-chord direction from the primary natural frequency, as in the wing-span direction. However, the deformation in the wing-chord direction is affected by the bending stiffness in the wing-chord direction. For reference, the wing-chord bending stiffness (*EI*_*yy*_) of a reference size wing is shown in Table 2. This bending stiffness was calculated using the structural analysis function of the CAD software (Inventor; Autodesk Inc.), using the same method as in the previous study (Combes and Daniel, 2003).

**TABLE 2:** Wing-chord bending stiffness of a reference wing.

Wing vein	Wing-chord bending stiffness *EI*_*yy*_ [ N ⋅ mm^2^]
ALL	1345.664
S	685.964
C	1524.736
SC	1442.470

### 2.2 Measurement of forces produced by a simple flap of wing

We experimented with measuring the force produced by the flapping motion of the wings we created. First, we describe the experimental setup, which is similar to a pendulum, that we used to measure the forces. Next, we describe the direction in which the force was generated by flapping the wings, which we measured during the study. We also provide details regarding the flapping device we utilized in the experiments. Finally, we describe normalization.

**FIGURE 4:**
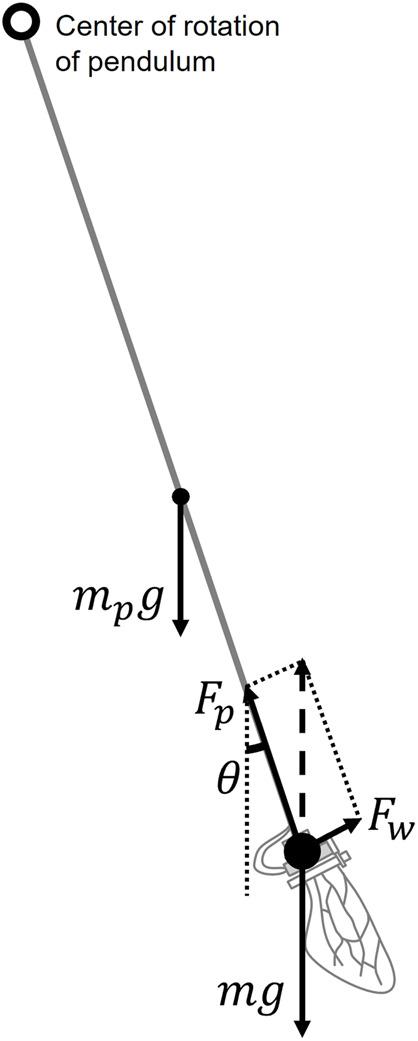
Relationship between the force *F*_*w*_ produced by the wing flapping, the gravity of the flapping device *mg*, the gravity of the pendulum rod *m*_*p*_ *g* and the force *F*_*p*_ produced by the pendulum device. The pendulum angle *θ* oscillates in a damped manner at the beginning of flapping, but when it converges, these forces reach a balance.

**FIGURE 5:**
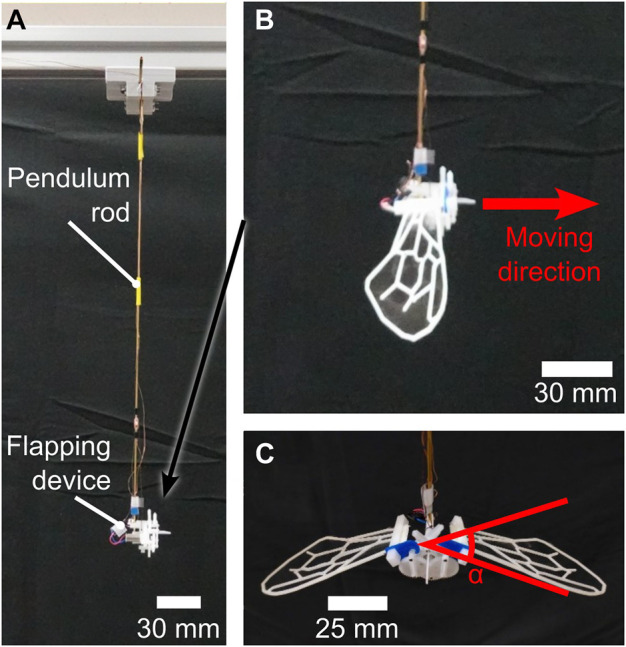
Pendulum and flapping device used in this study. **(A)** Overall view of the pendulum device without wings attached. **(B)** Close-up view of the flapping device with attached wings, from the side. **(C)** Close-up view of the flapping device with attached wings, from the front.

To investigate the force produced by the flapping of wings, we developed a pendulum-like experimental setup (Figure 5A) based on the swing test method of previous research (Nguyen et al., 2010). According to their previous study, this swing test method can perform the same measurement as the general measurement method using a load cell (Nguyen et al., 2010). A brass pipe was used as the rod of the pendulum, and a flapping device was attached to its tip. When the wings generate force by flapping, the pendulum tilts. The combined force generated by flapping (*F*_*w*_) and the pipe pulling the flapping device (*F*_*p*_) balance the gravity on the flapping device and the pendulum rod, where *F*_*w*_ is the force in the direction perpendicular to the flapping plane. Gravity on the pendulum rod occurs at the center of the pendulum rod. Therefore, by measuring the angle *θ* of the pendulum at this point of balance, the force (*F*_*w*_) generated by the flap was obtained using

$$F_{w}=\left(m+\frac{m_{p}}{2}\right)g\sin\theta ,$$ (3)

where *m* is the mass of the flapping device, *m*_*p*_ is the mass of the pendulum rod and *g* is the gravitational acceleration, as shown in Figure 4. We calculated the mass of the flapping device by combining the mass of the flapping device, excluding the wings (3.77 g), with double the mass of one wing including attachments as listed in Table 3. The mass of the pendulum rod was 1.90 g.

**TABLE 3:** Weight of wings. The unit is Gram. The values include the weight of attachment parts.

Wing vein	ALL	S	C	SC
Size [%]				
90	0.80	0.78	0.75	0.78
100	0.82	0.81	0.78	0.82
110	0.85	0.87	0.81	0.86
120	0.98	0.94	0.86	0.96
130	1.07	1.00	0.94	1.02
140	1.20	1.15	1.06	1.19
150	1.31	1.27	1.13	1.30

The pendulum was filmed using a camera (RX100VII; Sony Co.) during the experiment. The angle of the pendulum was measured by tracking the pendulum in the captured video using Kinovea (Charmant, 2021). The pendulum was tracked for more than 5 s starting approximately 2 s after the start of the flapping motion.

The pendulum performed a damped oscillation after the flapping motion started, until the forces due to gravity and flapping were balanced. The damped oscillation can be described as

$$\theta =Ae^{-\gamma t}\cos\left(\omega t+\alpha \right)+B.$$ (4)

Here, *t* is time and *A*, *γ*, *ω*, *α*, and *B* are constants, where *γ* is the damping coefficient and is greater than zero. When *t* approaches infinity and the forces are balanced, the angle *θ* converges to *B*. The time required for sufficient damping depends on the generated flapping force, which varies depending on the wing used. In this study, the balancing angle *B*, when the damping oscillation converges, was obtained by fitting Eq. 4 to the change in angle obtained from tracking, to standardize the time for video recording of the wing.

We investigated only the force generated in the wing-chord direction (*F*_*w*_ in Figure 4), following a previous study (Nan et al., 2017). During a single flap, the force perpendicular to the wing surface was considered to be almost canceled out by the down-stroke and the up-stroke. In addition, the force in the wing-span direction was considered to be canceled out by the left and right wings. The force generated in the wing-chord direction is considered the largest among the forces generated by the flapping of the wings; hence, only this force was measured in this study.

**FIGURE 6:**
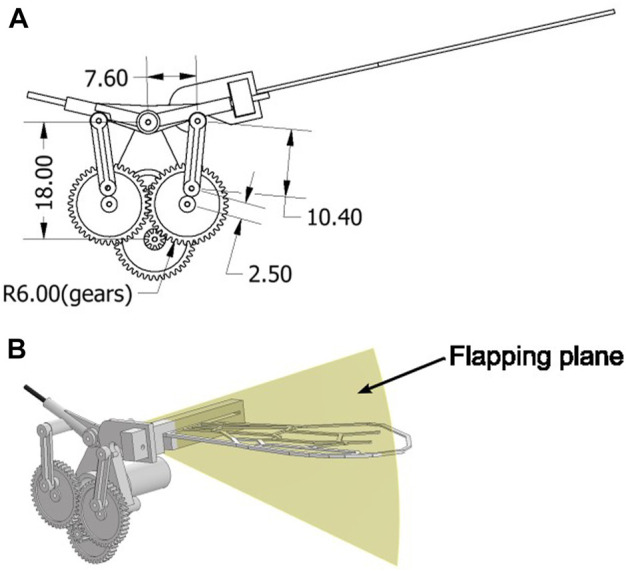
Diagram of the flapping device. **(A)** Detailed parameters of the gear crank of the flapping device. The unit is millimeter. **(B)** Flapping plane is shown in yellow and is perpendicular to the wing surface.

**FIGURE 7:**
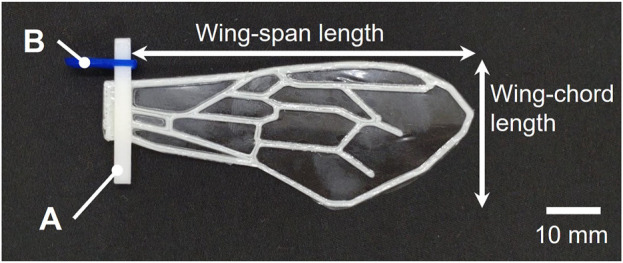
An example of a wing fabricated in this study, with measurement positions of wing-span length and wing-chord length indicated. The parts labeled **(A)** and **(B)** in the figure are for attaching the wing to the flapping device, with **(A)** made of POM and **(B)** made of PLA.

Figures 5B, C display the flapping device used in this study, which consists of a small motor and a plastic gear crank mechanism. Figure 6A shows the detailed parameters of the gear crank mechanism. Torque from the installed DC motor transmitted to the crank mechanism is amplified by a gear ratio of 1600/63:1. During the experiment, 5 V power was applied to the motor, generating a flapping motion at a frequency of approximately 32 Hz. This flapping frequency was measured using a camera (RX100VII; Sony Co.) and Kinovea (Charmant, 2021). The flapping device performed a simple to-and-fro motion with an angle (*α*) of approximately 39.69° (Figure 5C). The flapping angle was calculated from the crank mechanism parameters shown in Figure 6A. The flapping plane was perpendicular to the wing surface (Figure 6B). We attached the artificial wing, as previously described, to the flapping device using attachment parts. A polyoxymethylene (POM) (Figure 7A) was attached to the base of the artificial wing using adhesive, and a 3D printed polylactic acid (PLA) (Figure 7B) was hook-shaped and press-fitted into the POM. These two parts were used to secure the artificial wing to the flapping device. These components ensure that the wing base remains stationary, essentially preventing any rotation at the base. Owing to the wing’s flexibility, rotational deformation in the pitch direction is only observed at the wing tips.

Here, we describe the choice of the flapping frequency of the flapping device. The previous study (Liu and Aono, 2009) defines the Reynolds number in hovering flight as

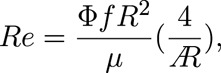

(5)
where Φ is the flapping amplitude, *f* is the flapping frequency, *R* is the wing string length, *μ* is the kinematic viscosity of air, and 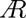 is the aspect ratio of the wing. In this study, the wing outline was designed based on the honeybee (*Apis mellifera*); thus, the aspect ratio 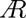 is the same as that of the honeybee. Therefore, to adjust the Reynolds number *Re* in this study to that of the actual honeybee, Φ*fR* ^2^ should be adjusted. According to the previous study (Ellington, 1984), Φ = 130 deg, *f* = 197 Hz, *R* = 9.8 mm for *Apis mellifera*. However, Φ = 39.69 deg, *R* = 57.254 mm for the reference wing in this study. Therefore, if Φ*fR* ^2^ is the same as that of the actual honeybee, the flapping frequency should be approximately *f* = 18.9 Hz. In this study, we adopted *f* = 32 Hz as a flapping frequency close to this value at which the motor used can stably generate sufficient torque.

The function of time *t* s for the flapping angle *φ* can be calculated from the gear-clank mechanism shown in Figure 6A as follows:

$$\begin{array}{ll} \varphi & =2\;atan\\ & \;\left(\frac{\frac{76}{1259}\sigma_{1}+\sqrt{{\left(\frac{76}{1259}\sigma_{1}-\frac{47842}{125}\right)}^{2}+\left(8\;\sigma_{2}+\frac{\sigma_{1}}{20}-\frac{591581}{10000}\right)\;\left(272\;\sigma_{2}-\frac{\sigma_{1}}{5}+\frac{2415581}{2500}\right)}-\frac{47842}{125}}{136\;\sigma_{2}-\frac{\sigma_{1}}{10}+\frac{2415581}{5000}}\right) \end{array},$$ (6)

where *σ*_1_ and *σ*_2_ are introduced to simplify the equation and are defined as follows:

$$\sigma_{1}=1259\;\sin\left(64\;\pi \;t\right)$$ (7)

$$\sigma_{2}=\cos\left(64\;\pi \;t\right).$$ (8)

From this equation, angular velocity and angular acceleration can be derived. The detailed derivation of the equation can be found in the supplementary materials.

Given that the parameters, such as wing size, are slightly different, and for comparison with the results of other studies, the force produced by the flapping of the wings must be normalized. Following the previous study (Balta et al., 2021), we define the coefficient of the force *C*_*f*_ produced by the flapping of wings as follows:

$$C_{f}=\frac{F_{w}}{N\frac{1}{2}\rho V_{ref}^{2}S},$$ (9)

where *N* is the number of wings, *ρ* is the air density, *S* is the wing area, and *V*_*ref*_ is the reference velocity. We used the maximum flapping speed for reference velocity, as in the previous study (Balta et al., 2021). Therefore, *V*_*ref*_ is defined as follows:

$$V_{ref}=2\pi fR\Phi ,$$ (10)

where *f* is the flapping frequency, *R* is the wing length, and Φ is the stroke amplitude.

## 3 Result

**FIGURE 8:**
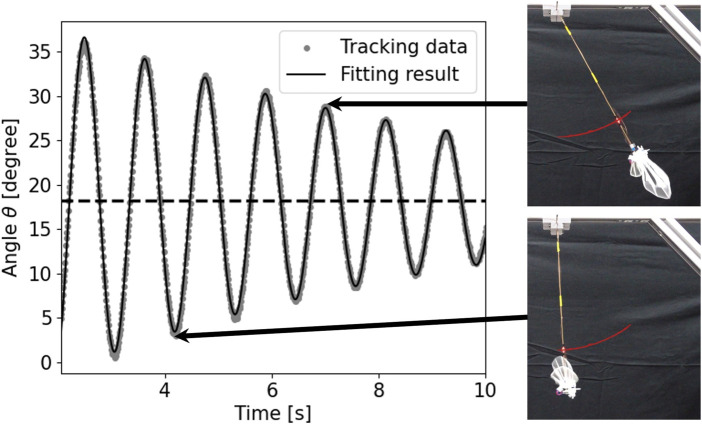
Time series data and snapshots of the pendulum device with wing vein type S and size of 150% of the reference wing. The horizontal axis represents time and the vertical axis represents the angle *θ* of the pendulum device. The dots in the graph represent angle data obtained by tracking, the solid line represents the result of fitting Eq. 4 to the tracking data, and the horizontal dotted line represents the value at the converged oscillation. The red line in the snapshot represents the tracking result including time before and after.

**FIGURE 9:**
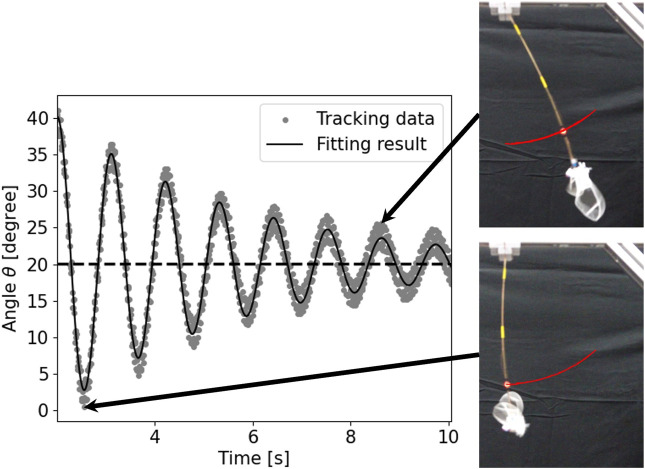
Time series data and snapshots of the pendulum device with wing vein type C and size of 140% of the reference wing. The horizontal axis represents time and the vertical axis represents the angle *θ* of the pendulum device. The dots in the graph represent the angle data obtained by tracking, the solid line represents the result of fitting Eq. 4 to the tracking data, and the horizontal dotted line represents the value at the converged oscillation. The red line in the snapshot represents the tracking result including time before and after.

As representative data, the tracking and fitting results of the pendulum angle are shown in Figure 8 for a wing with wing vein type S and wing size of 150% of the reference wing, and in Figure 9 for a wing with wing vein type C and wing size of 140% of the reference wing. The horizontal axis of the graph represents time, while the vertical axis indicates the angle of the pendulum device at each point in time. The dots in the graph represent the angle data obtained by tracking, the solid line shows the result of fitting Eq. 4 to the tracking data, and the horizontal dotted line displays the value at which the damping oscillation converges. When the wing vein type was S and the wing size was 150% of the reference wing, the convergence point was approximately 18.25°. Conversely, when the wing vein type was C and the wing size was 140% of the reference wing, the convergence point was approximately 20.11°. For some wings, including the wing with wing vein type C and a wing size of 140% of the reference wing, a minute vibration of the pendulum’s pipe was observed. In this study, the angle of convergence was calculated by fitting a damping function; this helped exclude errors in the measurement angle due to pipe vibration.

**FIGURE 10:**
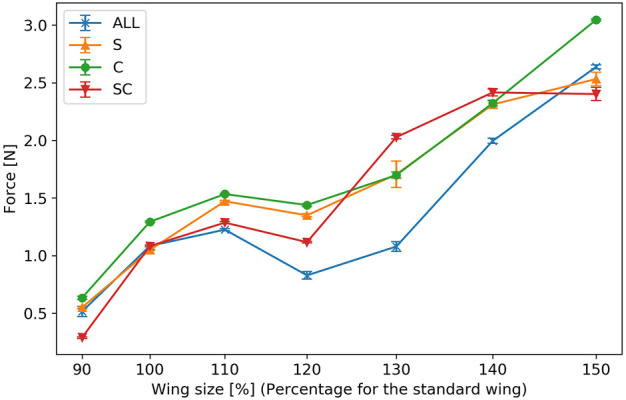
Force generated by wing flapping. The horizontal axis represents the wing size relative to a reference wing, and the vertical axis represents the force generated by wing flapping. The error bars indicate the standard errors.

For each artificial wing parameter, five sets of data were collected and the mean and standard error were obtained from the convergence of the pendulum device’s angle when using each wing. Figure 10 displays the results. The horizontal axis represents the length variation of the wing-span relative to the reference wing, while the vertical axis indicates the force produced by the flapping of the wings. The error bars show the standard error for each value. In all the experimental cases, except for wings with an area of approximately 1613 mm^2^ (130% of the reference wing size), wings with veins only in wing-chord direction generated more force than those with veins only in the wing-span direction. For a wing area of approximately 1613 mm^2^ (130% of the reference wing size), the crossed wing veins, which included both the wing-chord and wing-span veins, produced greater force. The force generated by wing flapping was not consistently proportional to the wing area for any wing vein type, and decreased for all vein types when the wing area was approximately 1375 mm^2^ (120% of the reference wing size).

**FIGURE 11:**
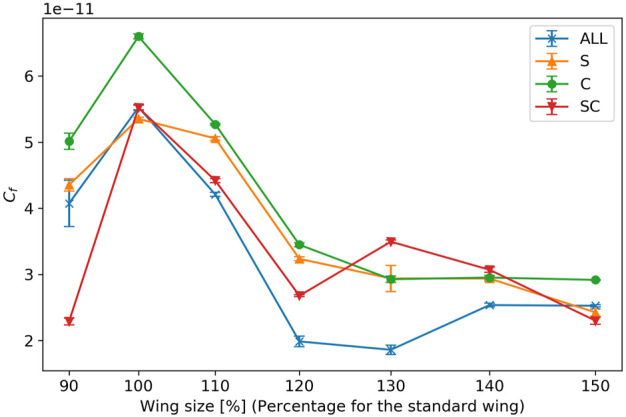
Force coefficient of wing flapping. The horizontal axis represents the wing size relative to a reference wing, and the vertical axis represents the force coefficient of wing flapping. The error bars indicate the standard errors.

Figure 11 shows the result of the force coefficient, which is the normalized force generated by the flapping of each wing. For all wing types, the force coefficient shows a maximum value when the wing area is 954 mm^2^ (100% of the reference wing size). The change in force coefficient between wing size of 120% and 150% of the reference wing size is smaller than that between 100% and 120% of the reference wing size.

## 4 Discussion

In this study, we focused on two types of simplified wing vein elements: the wing-chord directional veins and the wing-span directional veins, to investigate how the force generated by the wing changes with varying wing area. Wings with only wing-chord veins produced more force than those with only wing-span veins in all cases, except for the case when the wing area was approximately 1613 mm^2^ (130% of the reference wing size). The reason why wings with only wing-chord veins produced greater forces for almost all wing areas might be related to the difference in bending stiffness in the wing-chord direction, depending on the vein shape. The wings used in this study have thicker wing leading edges, similar to those found in insects. Consequently, the displacement of the wing leading edge is more constrained compared to that of the wing trailing edge. It is believed that this produces a displacement motion of the wing’s trailing edge around its leading edge. In the wing-chord direction, the wing may oscillate in a primary mode, with the wing’s leading edge acting as the fixed end of the cantilever. The wing veins extending from the wing leading edge to the wing trailing edge, i.e., the wing veins in the wing-chord direction, are believed to increase the bending stiffness in the wing-chord direction and limit the mode of deformation in the wing-chord direction to a primary mode. Wings without wing-chord veins may possess lower stiffness in the wing-chord direction compared to those with wing veins, potentially oscillate in second-order modes or higher and generating less force. The force produced by wing flapping generally increased with larger wing area. However, it was not always proportional.

The force tended to decrease when the wing area was approximately 1375 mm^2^ (120% of the reference wing size) for all the wing veins. In contrast, when the wing area was approximately 1613 mm^2^ (130% of the reference wing size), crossed veins containing both the wing-chord and wing-span veins generated more force. This suggests that, compared to wing-span veins, wing-chord veins tend to have a greater influence on the forces produced. However, some wing areas may require a combination of wing-chord and wing-span veins. In previous studies, wing flapping experiments on wings with leading edge wing veins and membranes (Ryu et al., 2019), as well as experiments focusing on wing stiffness (Lua et al., 2010), have been conducted to investigate the forces generated by the flapping of flexible wings. These studies suggest that flexible wings exhibit higher aerodynamic performance within a specific range of wing-chord directional flexibility. In the present study, the effect of changes in wing-span inertia was suppressed by keeping the wing-span natural frequencies the same. The results of this study may be attributed to the characteristics of the wing-span. The results drawn from this study, in conjunction with prior findings, indicate that the wing-chord flexibility of wing vein type S in the wing-chord direction was inadequate, whereas it was more suited for wing vein type C. In addition, the suitable wing vein design differed according to the wing area. This suggests that even wings made of the same material and flapping in the same mode may have different ranges of suitable flexibility depending on the wing area. Optimizing wing stiffness, as well as the wing area, should therefore be considered.

In this study, the relationship between the wing veins and the force generated by wing flapping was experimentally investigated using the flapping motion that is most commonly utilized in MAVs. The flapping robot has a mechanism that allows for passive pitch rotation of the wings in some previous studies (Dileo and Deng, 2009; Ma et al., 2013; Zou et al., 2016; Chen et al., 2019). However, the flapping robot does not have a mechanism that allows for passive pitch rotation of the wings in some other previous studies (De Croon et al., 2009; Gerdes et al., 2014; Sukvichai and Yajai, 2020). Because this study focuses on the performance of the simplest flapping robot, which results from the flexibility of the wings, experiments were conducted with the wing roots fixed so that pitch rotation would not occur. In addition, we selected the wing manufacturing method and flapping device that are easy to manufacture and use for flapping robots. The wing fabrication and flapping motion generation methods used in this experiment are practical and can be easily adapted for MAV fabrication. People can apply the data gained in this study to create actual MAVs.

This study was conducted to obtain experimental knowledge regarding the relationship between wing vein design and the forces produced by wing flapping. The results were consistent with previous research. This study simultaneously compared artificial and biological wing vein patterns and supported its consistent results with previous studies. Also, this study’s data is valuable for numerical simulations of the flapping motion of flexible wings. It is because simulating the flapping motion of a non-uniformly flexible wing that deforms during the flapping motion and the force generated by the flapping motion is challenging, as it involves the interaction between an elastic body and a fluid.

A possible avenue for future investigation is the impact of wing shape on the force generated by wing flapping. In this study, all wings had the same external shape, but previous research has shown that wing aspect ratio and other factors related to wing shape can influence the force generated during wing flapping (Nan et al., 2017). Therefore, it is possible that the effect of wing veins on wing flapping force may vary depending on specific wing shape. In addition, this study only investigated the effect of wing veins in the wing-chord direction on the force generated by wing flapping, and it was found that in six out of the seven different wing areas tested, wing veins only in the wing-chord direction produced either the greatest force or a force equivalent to the largest. However, the optimal number of wing veins in the wing-chord direction for producing the greatest force was not investigated in this study. An additional issue that needs to be investigated in the future is the effect of the number of wing veins. Moreover, when the wing area was approximately 1613 mm^2^ (130% of the reference wing size), crossed wing veins generated more force than wing-chord veins, but no numerical data were obtained to explain this. Measuring the shape of the wing surface during the flapping process will provide more insights in the future.

Importantly, the accuracy of the 3D printer used in this study is limited. The width of the printed wing veins is narrow and approaches the minimum limit of the 3D printer. This is particularly true for smaller wings, such as those that are 90% of the size of the reference wing, where thickness is also close to the minimum limit of the 3D printer because of the limited number of printing layers in the smallest part. This could result in variations in printing accuracy among different 3D printers. Additionally, the uniform enlargement and reduction of the wing size makes it difficult to determine which specific changes in wing size had greater impact on the relationship between the force produced by wing flapping and wing size. While the experimental results showed lower performance at specific wing area for all wing veins, it was not possible to identify which part of the wing size was responsible for this.

One limitation of this study is that it considered only robotic applications and did not consider the detailed body structure of insects. We considered this research as a study of artificial wings for use in flapping robots and did not aim to investigate the characteristics of actual insect wings. Therefore, the thickness of the wing was uniform except for the leading edge, in order to investigate the characteristics of the artificial wing in a manufacturing process that is easy to use. For this reason, the material is different from the material of actual insect wings, although it is effective as information when employed in a flapping robot. Additionally, we did not take into account the microstructure, the variation in the thickness of the wing veins, and the cavities inside the veins, which are found in the wings of actual insects. The structural characteristics of insect wings cannot be discussed from the results of this study.

## 5 Conclusion

In this study, we investigated how the forces generated by flapping change when the wing area is changed by focusing on two types of wing vein elements, namely, the simplified wing-chord directional veins and the simplified wing-span directional veins. The results showed that the wings with only wing-chord veins produced more force than wings with only wing-span veins in all the cases of the wings used in the experiment, except for those with a wing area of approximately 1613 mm^2^ (130% of the reference wing size). The force generated by wing flapping tended to increase with an increase in the wing area. However, it was not always directly proportional to the wing area, and decreased for all wing veins when the wing area was approximately 1375 mm^2^ (120% of the reference wing size). For the creation of a simple flapping MAV, a wing design with only wing-chord-directed wing veins may be advantageous. However, for specific wing sizes, it may be beneficial to add wing veins in the wing-span direction.

## Data Availability

The raw data supporting the conclusion of this article will be made available by the authors, without undue reservation.
