# Supplementary material for: Effect of incorporating wing veins on soft wings for flapping micro air vehicles
Source: Front Robot AI. 2023 Aug 7;10:1243238. doi: 10.3389/frobt.2023.1243238 (PMC10440695; doi:10.3389/frobt.2023.1243238)
Supplement: Supplementary file 1 [file DataSheet1.PDF]

Supplementary Material

1 CALCULATION OF FLAPPING SPEED BASED ON CRANK MECHANISM

In this section, we calculate the flapping angle equation for the time of the flapping device. Here, we consider the crank mechanism of one of the flapping devices. Figure S1A shows the crank mechanism of one side of the flapping device. Point O is the central point of the flapping, and point A is the center of the gear rotated by the motor. The motion of the motor causes link AB to rotate and link OC to move up and down. The rotation of link AB is converted into the vertical motion of link OC by link BC.

Now, we define the origin at point O, the X-axis as horizontal to the ground and toward the wingtip, and the Y-axis as vertical to the ground and upward. Link OA is fixed, so the position of point A is unchanged. As shown in Figure S1B, we set the length of link AB to be l_{AB}, link BC to be l_{BC}, and link OC to be l_{OC}. We define ψ as the angle that link AB makes with the X-axis, i.e., the angle of rotation of link AB. We define φ as the angle linking OC with the X-axis, i.e., the flapping angle. In both angles, the counterclockwise rotation is positive. In this study, we rotated link AB at a constant velocity of 32 Hz. Therefore, if the initial angle at ψ is 0, the φ after t seconds is

$$\psi = \frac{2\pi}{32}t. \tag{S1}$$

When the position of point A is (x_A, y_A), the position of point B is (x_B, y_B), and the position of point C is (x_C, y_C), we have

$$\begin{aligned}(x_B,\, y_B) &= (x_A + l_{AB}\cos\psi,\, y_A + l_{AB}\sin\psi) &\text{(S2)}\\ (x_C,\, y_C) &= (l_{OC}\cos\varphi,\, l_{OC}\sin\varphi). &\text{(S3)}\end{aligned}$$

Since the length of the link BC is constant, it follows that

$$\begin{aligned}l_{BC}^2 &= (x_C - x_B)^2 + (y_C - y_B)^2\\ &= (l_{OC}\cos\varphi - x_A - l_{AB}\cos\psi)^2 + (l_{OC}\sin\varphi - y_A - l_{AB}\sin\psi)^2\\ &= l_{OC}^2 + l_{AB}^2 + {x_A}^2 + {y_A}^2\\ &\quad -2l_{OC}x_A\cos\varphi + 2l_{AB}x_A\cos\psi - 2l_{OC}l_{AB}\cos\varphi\cos\psi\\ &\quad -2l_{OC}y_A\sin\varphi + 2l_{AB}y_A\sin\psi - 2l_{OC}l_{AB}\sin\varphi\sin\psi. \end{aligned} \tag{S4}$$

When $a = \tan\frac{\varphi}{2}$, we have

$$\begin{aligned}\cos\varphi &= \frac{1-a^2}{1+a^2} &\text{(S5)}\\ \sin\varphi &= \frac{2a}{1+a^2}. &\text{(S6)}\end{aligned}$$

By substituting this into the equation S4, we obtain

$$\begin{aligned} l_{BC}^2 &= l_{OC}^2 + l_{AB}^2 + {x_A}^2 + {y_A}^2 \\ &\quad -2l_{OC}x_A\frac{1-a^2}{1+a^2} + 2l_{AB}x_A\cos\psi - 2l_{OC}l_{AB}\frac{1-a^2}{1+a^2}\cos\psi \\ &\quad -2l_{OC}y_A\frac{2a}{1+a^2} + 2l_{AB}y_A\sin\psi - 2l_{OC}l_{AB}\frac{2a}{1+a^2}\sin\psi. \end{aligned} \tag{S7}$$

Multiplying both sides by $(1+a^2)$, we get

$$\begin{aligned} l_{BC}^2(1+a^2) &= (l_{OC}^2 + l_{AB}^2 + {x_A}^2 + {y_A}^2)(1+a^2) \\ &\quad -2l_{OC}x_A(1-a^2) + 2l_{AB}x_A\cos\psi(1+a^2) - 2l_{OC}l_{AB}(1-a^2)\cos\psi \\ &\quad -2l_{OC}y_A(2a) + 2l_{AB}y_A\sin\psi(1+a^2) - 2l_{OC}l_{AB}(2a)\sin\psi. \end{aligned} \tag{S8}$$

This can be rewritten as follows:

$$\begin{aligned} 0 &= (l_{BC}^2 - l_{OC}^2 - l_{AB}^2 - {x_A}^2 - {y_A}^2 - 2l_{OC}x_A - 2l_{AB}x_A\cos\psi - 2l_{OC}l_{AB}\cos\psi - 2l_{AB}y_A\sin\psi)a^2 \\ &\quad +(4l_{OC}y_A + 4l_{OC}l_{AB}\sin\psi)a \\ &\quad +l_{BC}^2 - l_{OC}^2 - l_{AB}^2 - {x_A}^2 \\ &\quad -{y_A}^2 + 2l_{OC}x_A - 2l_{AB}x_A\cos\psi + 2l_{OC}l_{AB}\cos\psi - 2l_{AB}y_A\sin\psi. \end{aligned} \tag{S9}$$

Where α, β, and γ are defined as follows:

$$\begin{aligned} \alpha &= l_{BC}^2 - l_{OC}^2 - l_{AB}^2 - {x_A}^2 - {y_A}^2 \\ &\quad -2l_{OC}x_A - 2l_{AB}x_A\cos\psi - 2l_{OC}l_{AB}\cos\psi - 2l_{AB}y_A\sin\psi \end{aligned} \tag{S10}$$

$$\beta = 4l_{OC}y_A + 4l_{OC}l_{AB}\sin\psi \tag{S11}$$

$$\begin{aligned} \gamma &= l_{BC}^2 - l_{OC}^2 - l_{AB}^2 - {x_A}^2 - {y_A}^2 \\ &\quad +2l_{OC}x_A - 2l_{AB}x_A\cos\psi + 2l_{OC}l_{AB}\cos\psi - 2l_{AB}y_A\sin\psi, \end{aligned} \tag{S12}$$

the equation can be solved by using the quadratic formula:

$$a = \frac{-\beta \pm \sqrt{\beta^2 - 4\alpha\gamma}}{2\alpha}. \tag{S13}$$

Because of $a = \tan\frac{\varphi}{2}$,

$$\varphi = 2\tan^{-1}\left(\frac{-\beta \pm \sqrt{\beta^2 - 4\alpha\gamma}}{2\alpha}\right). \tag{S14}$$

Since φ ranges from $-\frac{\pi}{2} \leq \varphi \leq \frac{\pi}{2}$ because of the structure of the mechanism, φ is limited to the following:

$$\varphi = 2\tan^{-1}\left(\frac{-\beta - \sqrt{\beta^2 - 4\alpha\gamma}}{2\alpha}\right). \tag{S15}$$

The parameters used in the above calculations take the values of table S1 in this study.

Symbol	Value [mm]
x_a	6.00
y_a	-12.59
l_{AB}	2.50
l_{BC}	10.40
l_{OC}	7.60

Table S1. Values of each variable in this study.

Substituting the values in equation S1 and table S1 into the equations S10, S11, S12, and S15, we obtain

$$\varphi = 2\,\mathrm{atan}\left(\frac{\frac{76}{1259}\sigma_1 + \sqrt{\left(\frac{76}{1259}\sigma_1 - \frac{47842}{125}\right)^2 + \left(8\,\sigma_2 + \frac{\sigma_1}{20} - \frac{591581}{10000}\right)\left(272\,\sigma_2 - \frac{\sigma_1}{5} + \frac{2415581}{2500}\right)} - \frac{47842}{125}}{136\,\sigma_2 - \frac{\sigma_1}{10} + \frac{2415581}{5000}}\right), \tag{S16}$$

where σ_1 and σ_2 are introduced to simplify the equation and are defined as follows:

$$\sigma_1 = 1259\sin(64\,\pi\,t) \tag{S17}$$

$$\sigma_2 = \cos(64\,\pi\,t)\,. \tag{S18}$$

Now we obtained the change of φ with respect to time.

Using the calculation software (MATLAB; MathWorks, Inc.), the angle φ, angular velocity $\dot{\varphi}$ and angular acceleration $\ddot{\varphi}$ are calculated as shown in Figure S2, S3, and S4, respectively.

1.1 Figures and Table

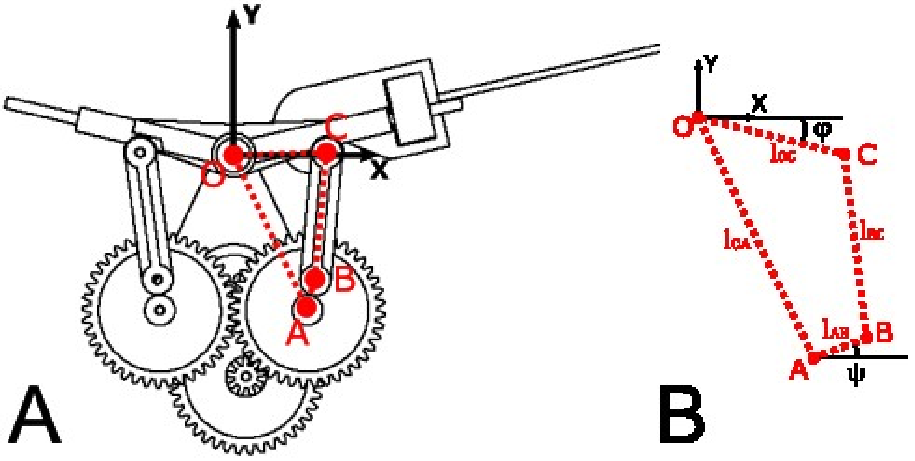

Figure S1. The flapping crank mechanism in this study. (A) Correspondence between the flapping device and the crank. (B) Definition of each point and angle in the crank mechanism.

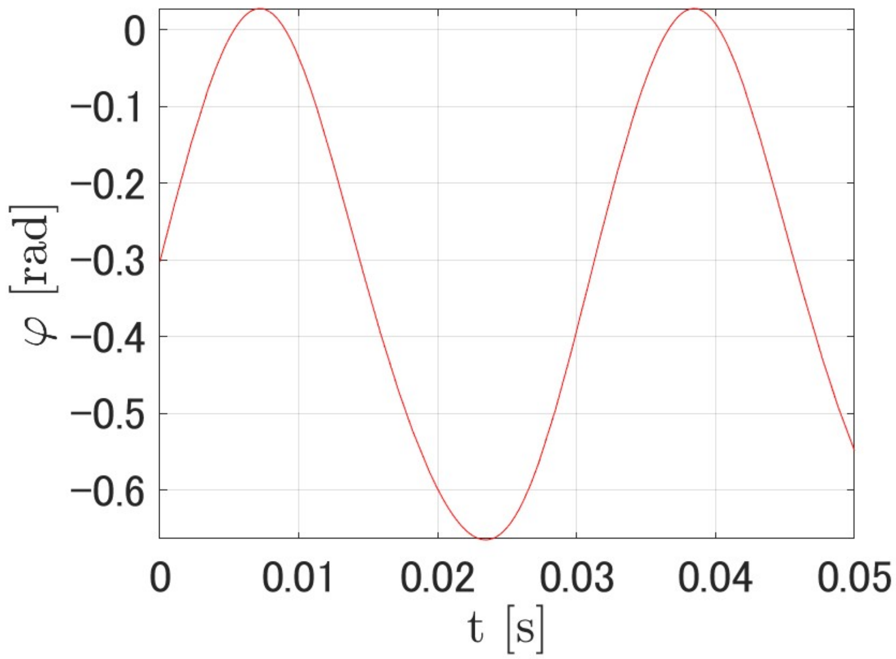

Figure S2. Graph of flapping angle φ relative to time t.

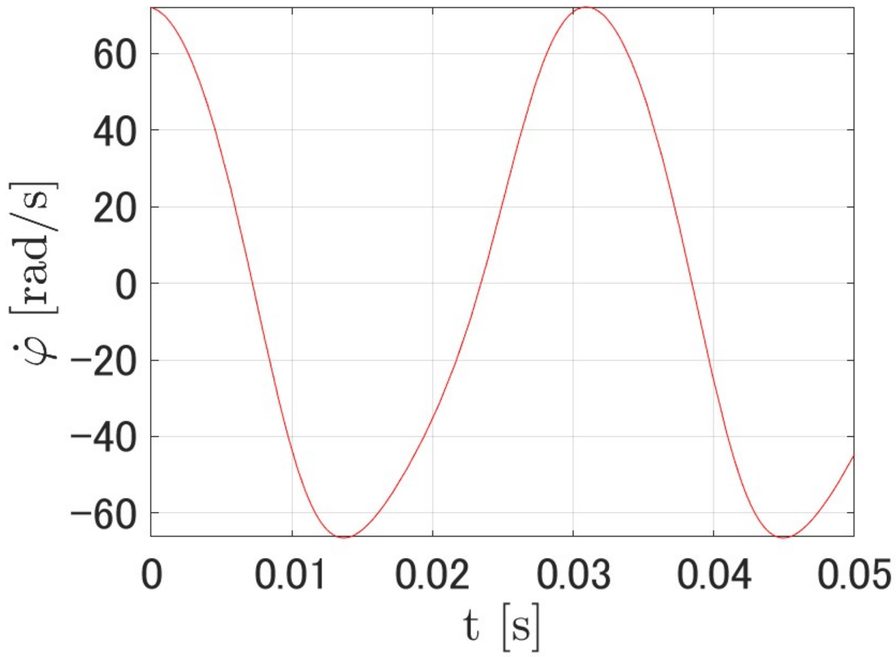

Figure S3. Graph of flapping angle velocity φ' relative to time t.

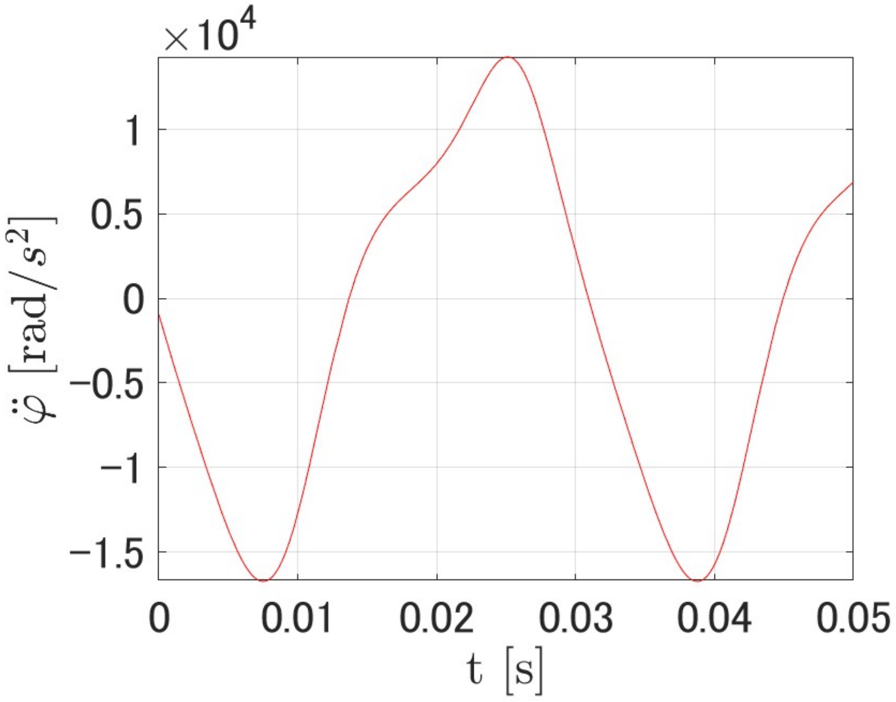

Figure S4. Graph of flapping angle acceleration φ'' relative to time t.
